# Effects of Prenatal Herbal Methionine Supplementation on Growth Indices, Onset of Puberty, Blood Metabolites, and Fertility of Alpine Doelings †

**DOI:** 10.3390/biology14030237

**Published:** 2025-02-26

**Authors:** Venancio Cuevas-Reyes, Jorge Flores-Sánchez, Esau Ramírez de la Cruz, Juan M. Vázquez-García, Luisa E. S. Hernández-Arteaga, Reagan Sims, Jaime M. Cavazos-Galindo, Miguel Mellado, César A. Rosales-Nieto

**Affiliations:** 1 Instituto Nacional de Investigaciones Forestales, Agrícolas y Pecuarias, Campo Experimental Valle de México, Texcoco 56250, Mexico; cuevas.venancio@inifap.gob.mx; 2 Laboratorio de Especialidades Médicas, Facultad de Medicina, Universidad Autónoma San Luis Potosí, San Luis Potosí 78290, Mexico; jorge.flores@uaslp.mx; 3 Facultad de Agronomía y Veterinaria, Universidad Autónoma San Luis Potosí, San Luis Potosí 78321, Mexico; chapu_k13@hotmail.com (E.R.d.l.C.); manuel.vazquez@uaslp.mx (J.M.V.-G.); socorro.hernandez@uaslp.mx (L.E.S.H.-A.); 4 Department of Agricultural Sciences, Texas State University, San Marcos, TX 78666, USA; r_sims@txstate.edu; 5 Centro de Fomento Ganadero Vallecillo, Universidad Autónoma de Nuevo León, Vallecillo 65415, Mexico; jaime_cavazos76@hotmail.com; 6 Departamento de Nutrición Animal, Universidad Autónoma Agraria Antonio Narro, Saltillo 25315, Mexico; miguel.mellado@uaaan.mx

**Keywords:** doeling, reproduction, growth, age and liveweight at puberty, Alpine

## Abstract

This study investigated the effects of prenatal herbal methionine supplementation on growth, puberty onset, reproductive efficiency, and blood metabolites in first-generation G1 Alpine doelings. Sixty pregnant multiparous goats (G0), each with at least three parturitions, were assigned during the last trimester of pregnancy to either herbal methionine (H-MET-G0; 1% Optimethione^®^; n = 30) or a control group (CTL-G0; n = 30). Only female offspring (G1 doelings) were studied (H-MET-G1:19; CTL-G1:25) from weaning (45 days old) to 10 months (first breeding). G1 doelings were weighed and monitored weekly for metabolites, IGF-1, insulin, and progesterone. Ovulation of the G1 doelings was induced using vasectomized bucks, followed by breeding with intact bucks. Postweaning growth, age at puberty (H-MET-G1:216 vs. CTL-G1:229 days), and live weight at puberty (H-MET-G1:20.5 vs. CTL-G1:21.0 kg) did not significantly differ (*p* > 0.05). Age at puberty and conception were negatively correlated with live weight (*p* < 0.001). First-cycle conception rates were 81% in CTL-G1 and 66% in H-MET-G1, with similar days to conception (*p* > 0.05). The number of pregnant doelings and the number of kids in utero did not significantly differ (*p* > 0.05) between treatments. Plasma IGF-1 and insulin levels were significantly higher (*p* < 0.001) in H-MET-G1 doelings, whereas plasma metabolites related to nutrition showed no differences between groups (*p* > 0.05). Prenatal herbal methionine supplementation had no carry-over effect on growth, puberty onset, or reproductive efficiency. Age at puberty and conception were negatively correlated with live weight and positively correlated with IGF-1 and insulin levels.

## Simple Summary

Our study investigated the effects of prenatal supplementation with herbal methionine on postweaning growth, puberty onset, and reproductive efficiency in purebred Alpine doelings. The results indicate that while supplementation increased IGF-1 and insulin levels, it did not lead to improved growth, earlier puberty, or enhanced reproductive performance. However, we found a strong negative correlation between body weight and the age at puberty and conception, suggesting that body weight is a more significant factor in reproductive maturity than prenatal methionine supplementation. These findings highlight the importance of growth management for goat producers aiming to optimize reproductive performance.

## 1. Introduction

During gestation, maternal energy distribution shifts, reducing placental energy consumption. As pregnancy progresses, metabolizable energy utilization for fetal development becomes more efficient [1]. In late gestation, dams often decrease their voluntary feed intake due to the rapid growth of the fetoplacental unit, which occupies more space in the abdomen and restricts gastrointestinal capacity [2]. This observation aligns with the onset of lactogenesis, and as pregnancy and lactogenesis coincide, the fetus becomes sensitive to maternal nutrition and competes with milk synthesis for nutrients [3,4]. This competition forces the fetus to adapt to nutritional challenges, impacting its growth and influencing processes like prenatal muscle and fat development and birth weight [5,6].

Research consistently demonstrates the significant influence of maternal nutrient intake on offspring’s postnatal development and health [7,8]. Nutritional stress during gestation can impair fetal development, leading to metabolic and reproductive dysfunctions in offspring. These stressors can lead to long-term consequences, adversely impacting the offsprings’ metabolic and reproductive performance by impairing the somatotropic axis’s activation [9,10,11] and the ability of subcutaneous adipose tissue to expand [12,13,14]. Both pathways are involved in the mammals’ reproductive axis. Insulin and insulin-like growth factor 1 (IGF-1) regulate the metabolism and control the body’s growth and puberty’s onset by activating kisspeptin and GnRH neurons [15,16]. Similarly, adipose tissue secretes leptin, which plays a permissive role in the metabolic onset of puberty by activating kisspeptin neurons and regulating reproductive efficiency [17,18]. Farm animals that experience delayed growth and reduced fat tissue often have delayed puberty, which can negatively impact their reproductive performance [19,20]. By contrast, fast-growing females accumulate more body reserves, secrete more leptin, attain puberty sooner, and are more fertile than slow-growing females [18]. In gilts and heifers, postnatal methionine supplementation accelerates the onset of puberty [21,22].

Methionine is an essential amino acid that serves as the precursor of S-adenosylmethionine (SAM) and is involved in diverse cellular processes and deoxyribonucleic acid methylation (DNA; [23,24]). Prenatal nutritional challenges and fluctuations in methionine dysregulate gene expression, fetal growth development [25,26,27], and postnatal growth [28,29]. Prenatal methionine supplementation in different animal species improved methylation processes and increased lipid, energy metabolism, and IGF pathways that resulted in increased fetal weight, birth weight, and weaning weight in swine [30], goats [31], cattle [32], and sheep [33]. Prenatal methionine supplementation may have a postnatal carryover effect as those newborns who were heavier at birth were heavier at weaning [30,32,33]. While the impact of growth factors, adipose, and muscle tissue on the onset of puberty and reproductive efficiency has been well characterized [19,20,34], the consequences of prenatal methionine supplementation on postweaning growth, onset of puberty, and reproductive efficiency of the subsequent generation remain poorly understood. Therefore, in the present study, we investigated whether prenatal herbal methionine supplementation in late gestation impacts metabolic profile, postweaning growth performance, metabolic parameters of offspring, and their onset of puberty and reproductive performance. We hypothesize that prenatal herbal methionine supplementation will improve postweaning growth, enhance metabolic profile, accelerate puberty onset, and increase reproductive performance in female goats.

## 2. Materials and Methods

The study was conducted at the Facultad de Agronomia y Veterinaria from the University of San Luis Potosi, Mexico (22°13′ N, 100°51′ W). The Institutional Animal Care and Use Committee (C19-FAI-05-86.86, 511-6/2019.-8024; 511-6/2019.-12305) approved all procedures in this study, consistent with international guidelines [35] and national guidelines [36] for the care and use of laboratory animals.

### 2.1. Experimental Design

To investigate the effects of prenatal herbal methionine (Optimethionine^®^; Nuproxa, Queretaro, Mexico) supplementation during the last trimester of pregnancy on offspring growth, development, puberty onset, and first pregnancy, 60 multiparous pregnant purebred Alpine goats (G0) with at least three parities and their female progeny (doelings; G1; n = 38) from the university pen-raised herd were included in the experiment. G0 dams were assigned to either a control group (CTL-G0; no methionine) or a treatment group (H-MET-G0; herbal methionine). Blocking was done at enrollment based on gestational age (100 ± 0.5 days) and body weight (50.4 ± 6.6 kg). Management of the dams (G0) and newborns (G1) at birth and up to weaning was previously reported [31].

**Figure 1:**
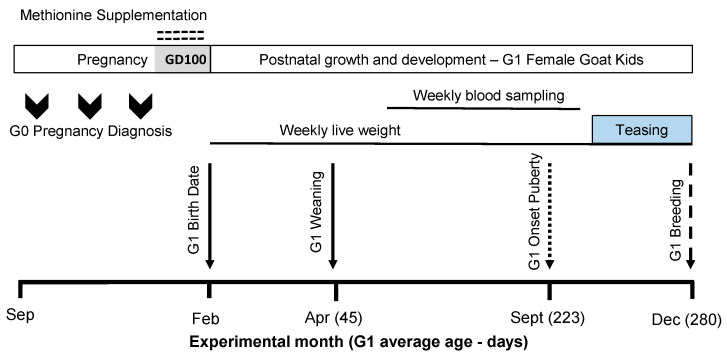
The scheme describing the experimental design used, including herbal methionine (H-MET-G0; Optimethione^®^) or not (CTL-G0) in the maternal diet on the estimated gestational date 100 up to parturition. The shaded area (GD100) during the pregnancy period represents when herbal methionine supplementation was started. Only female offspring (G1 doelings) were studied (H-MET-G1: n = 19; CTL-G1: n = 25) from weaning (45 days old) to 10 months (first breeding). The dotted-line arrow indicates the estimated onset of puberty (see details below). The dashed-line arrow marks the introduction of intact fertile bucks for breeding.

In brief, pregnant goats in the H-MET-G0 treatment were fed once daily with herbal methionine (Optimethionine^®^) during the last 50 days of pregnancy (Figure 1; [31]). Daily, each dam received 3% based on live weight (~13.5 g/goat/d) with 50 g of commercial balanced feed (Nu-3^®^ Ganado Lechero, 18% CP) in the milking parlor to ensure adequate methionine ingestion. The milking parlor accommodated 12 goats at a time, allowing for individual supplementation. The supplemented amount was adjusted weekly per live weight [31]. The control group (CTL-G0) received 50 g of commercial balanced feed without the H-MET supplement.

### 2.2. Maternal and Kids’ Diet

As previously described [31], a diet was designed to reflect the conditions experienced by goats in semiarid regions of northern Mexico (i.e., extensive management and undernutrition during pregnancy [37,38]). The maternal diet consisted of maize silage, alfalfa hay, and oats hay to satisfy maintenance requirements (1.35 kg dry matter [DM], 1.76 Mcal of metabolizable energy [ME], 69 g of crude protein [CP]; [39]). Afterward, dams received a diet that met the nutritional requirements for lactation (1.65 kg DM, 2.97 Mcal ME, 133 g CP; [39]). Drinking water was provided ad libitum throughout the trial. The total daily diet was divided into two equal portions, provided in the morning and afternoon during both periods. Feed was offered in a fence-line feeder with sufficient space to allow all animals to eat simultaneously, minimizing competition for space and feed access.

From birth to weaning (45 days), G1 goat kids (males (45) and females (46) and singletons (25) and twins (66); [31]) remained with their respective dams and had unrestricted access to both milk and the solid diet provided to their dams. After weaning, only female goat kids continued in the experiment (G1 doelings; n = 38; H-MET-G1: n = 19; CTL-G1: n = 25). Doelings from both treatments were housed together in a pen with enough space (head/1.5 m^2^). From weaning until their first breeding, G1 doelings received a diet formulated to meet the nutritional requirements for growing female goats with limited daily weight gain (Table 1 [39]). This diet was designed to reflect the semiarid conditions in northern Mexico and similar environments, characterized by variable and limited rainfall and sparse vegetation [38]. The diet was based on alfalfa hay, oat hay, and maize silage and was provided twice daily (half in the morning and half in the afternoon). The nutritional composition of the diet (Table 1) was assessed by wet chemistry methodologies (AGROLAB Mexico S.A de C.V, Durango, Mexico). Before their first breeding (December–January; information below), doelings were dewormed with 50 mg closantel (Closantil 5%^®^, Chinoin, Ciudad de México, Mexico) and received an intramuscular injection of Vigantol^®^ (Bayer; Ciudad de México, Mexico; 500,000 IU of VitA, 75,000 IU of VitD3, and 50 mg of VitE). Goats had water ad libitum and free access to mineral blocks.

### 2.3. Newborn Management and Growth

**Figure 2:**
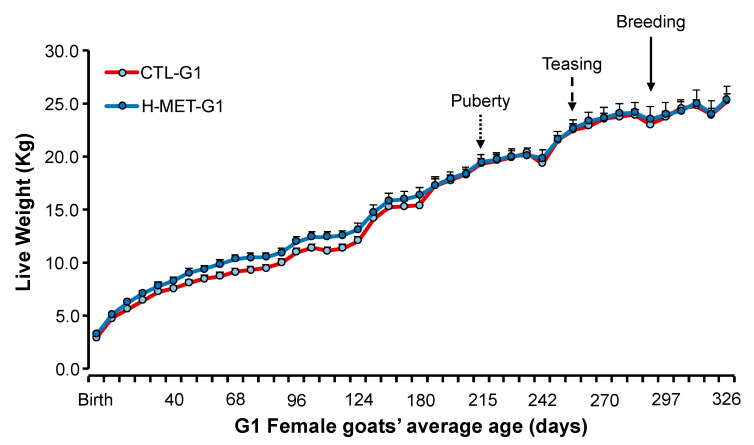
Live weight (mean ± SEM) from birth (February) to 10 months of age (end of January) in doelings born to dams that received herbal methionine (Optimethione^®^; H-MET-G0) or did not (CTL-G0) during the last trimester of pregnancy. The dotted-line arrow indicates the estimated onset of puberty. The dashed-line arrow marks the introduction of vasectomized bucks to induce ovulation. The solid-line arrow denotes the introduction of intact fertile bucks for breeding.

As previously described [31], gender, live weight, and litter size were recorded at birth. From birth to weaning (45 d), G1 goat kids were vaccinated with Clostri-10^®^ (Lapisa. La Piedad, Mexico). Male G1 kids were sold at weaning, and only female G1 kids (n = 38) were retained for the next phase of the experiment. Female G1 kids were housed together in a single pen and fed the diet described in Table 1. To assess female progeny growth (CTL-G1: n = 21; H-MET-G1: n = 17), live weights were recorded weekly from birth until the first breeding (9 months of age; Figure 2) using a mobile scale with a 200 kg capacity and a precision of 0.05 kg (Torey^®^).

### 2.4. Plasma Progesterone and Puberty

The age of puberty was determined based on the criteria established by Zarazaga et al. [40] and Espinoza-Flores et al. [41], whereby puberty was defined as the presence of plasma progesterone concentration ≥0.5 ng/mL in two consecutive blood samples. The live weight recorded closest to this date was considered the live weight at puberty. Weekly plasma progesterone concentrations (see details below) were measured using an automated IMMULITE^®^ 1000 progesterone immunoassay kit (catalog number LKPW1; Siemens Healthcare, New York, NY, USA). It had a calibration range of 0.20 to 40 ng/mL and a sensitivity of 0.46 ng/mL.

### 2.5. Reproductive Performance

Forty days before breeding, G1 doelings were teased by a vasectomized buck to induce estrus [42]. Subsequently, doeling’s pregnancy was achieved through natural mating with a purebred, experienced, intact buck over a 42 day-period (2 estrous cycles) from December to January (22°N and 100°W; Figure 1). The buck/doeling ratio was 1:38. Fertility proven bucks were used. The kidding date and birth weight of the G2 progeny were recorded. Using these data, the G1 conception date and days to conception after the introduction of fertile bucks were estimated by subtracting 150 days (goat’s gestational length [43]) from the G2 birth date. Fertility (percentage of doelings pregnant per 100 doelings mated), litter size (number of fetuses per pregnant doeling), and reproductive rate (number of fetuses per 100 doelings exposed to fertile bucks) were calculated based on the number of G2 kids born (singletons and twins).

### 2.6. Serological Analysis

Weekly blood samples were collected from all G1 doelings via jugular venipuncture before feeding, from May (on average 53 days old) to October (on average 206 days old). Samples were drawn into 10 mL collection tubes containing EDTA (BD Vacutainer, Preanalytical Solutions, Franklin Lakes, NJ, USA) and kept on ice until plasma separation by centrifugation (3000 rpm for 15 min). The isolated plasma was then stored at −20 °C until analysis. Plasma samples were assayed for total protein, urea, cholesterol, and glucose. For IGF-1 and insulin analysis, plasma from all doelings within each treatment group was pooled into two composite samples per date. Analytical procedures are detailed below.

Before analysis, the samples were thawed to room temperature and tested in duplicate. Quantification was performed internally for each duplicate, and the resulting values were averaged. The coefficient of variation was calculated, and all reported concentrations had a coefficient of variation below 10%.

### 2.7. Metabolites and Metabolic Hormones

For all analyses, quality control samples were considered acceptable if the coefficient of variation was below 10% for each level and if replicate measurements fell within the manufacturer’s specified range. Plasma urea and total protein concentrations were determined using an automated spectrophotometer MINDRAY BS-200 chemical analyzer, which was pre-calibrated with a calibrator, control serum levels I and II, and commercial reagents (Spinreact Urea-LQ^®^, Spinreact Total Protein^®^). Its sensitivity was 1 mg/dL. Plasma cholesterol and glucose concentrations were measured using an automated analyzer A25 BioSystems, which was also pre-calibrated with a calibrator, control serum levels I and II, and commercial reagents (Cholesterol BioSystems and Glucose BioSystems). The spectrophotometer’s sensitivity was 0.9 mg/dL for cholesterol and 1.6 mg/dL for glucose. Plasma IGF-1 concentrations were quantified using an automated IMMULITE^®^ 1000 IGF-1 immunoassay kit (catalog number LKIGF1; Siemens Healthcare, New York, NY, USA) with a calibration range of 15 to 1000 ng/mL and 14.4 ng/mL sensitivity. Plasma insulin concentrations were measured using an automated IMMULITE^®^ 1000 insulin immunoassay kit (catalog number LKIN1; Siemens Healthcare, New York, NY, USA) with a calibration range of 2 to 300 μIU/mL and a sensitivity of 2 μIU/mL.

### 2.8. Statistical Analysis

Individual animals were experimental units. G1 data were analyzed using the SAS statistical package (version 9.4) [44], following a completely randomized design. Data normality was assessed using the Shapiro–Wilk test (PROC UNIVARIATE). Urea, total protein, cholesterol, glucose, IGF-1, insulin, and progesterone were analyzed using linear mixed model procedures (PROC-MIXED). Treatment was the fixed effect. Live weight (across time) was included as a covariate. The sampling dates were included as repeated measures, and animal ID was included as a random effect.

Age and live weight at puberty, age and live weight at the start of the mating period, and age and live weight at conception were analyzed using the PROC-MIXED procedure. Treatment was the fixed effect in the model. Live weight (across time), plasma urea, total protein, cholesterol, and glucose were included as co-variables. The sampling dates were included as repeated measures, and animal ID was included as a random effect. For age and live weight at conception, age and live weight at the start of the mating period were included as co-variables.

Reproductive variables were calculated based on the number of G2 kids born. Puberty, fertility (pregnant or not) rate, and litter size (number of kids on the ground) were analyzed using the generalized linear mixed model procedures with a binomial distribution and logit link function (PROC-GLIMMIX). In each analysis, treatment was the fixed effect. For fertility and litter size, age and live weight at the start of mating were included independently as covariates. Data for reproductive rate (number of kids born per total number of mated doelings) were analyzed using the generalized linear mixed model procedures with a multinomial distribution and logit link function (PROC-GLIMMIX). The same fixed effect and covariates were used for the analysis of fertility.

Correlations among age, live weight at puberty, breeding period, and metabolic markers (protein, urea, cholesterol, glucose) were analyzed using PROC GLM with the MANOVA option, which allows removal of major fixed effects. A correlation value of >0.7 to near 1.0 was considered strong. A correlation value between 0.4 and 0.6 was considered moderate. A correlation value < 0.4 to near 0.0 was considered weak. All 2-way interactions among the fixed effect and weeks of sampling were included in each model, and non-significant (*p* > 0.05) interactions were removed from the model. The fertility and reproductive rate data are presented as logit values and back-transformed percentages. All continuous data are presented as mean ± SEM.

## 3. Results

### 3.1. G1 Birth Weight and Postnatal Growth

Birth weight differed between treatments (*p* < 0.01), but weaning weight did not (*p* > 0.05; Table 2). Live weight from birth to their first breeding was not significantly different between treatments (*p* > 0.05; Figure 2). Live weight across the experiment differed (*p* < 0.001; Figure 2).

### 3.2. G1 Age and Live Weight at Puberty

**Figure 3:**
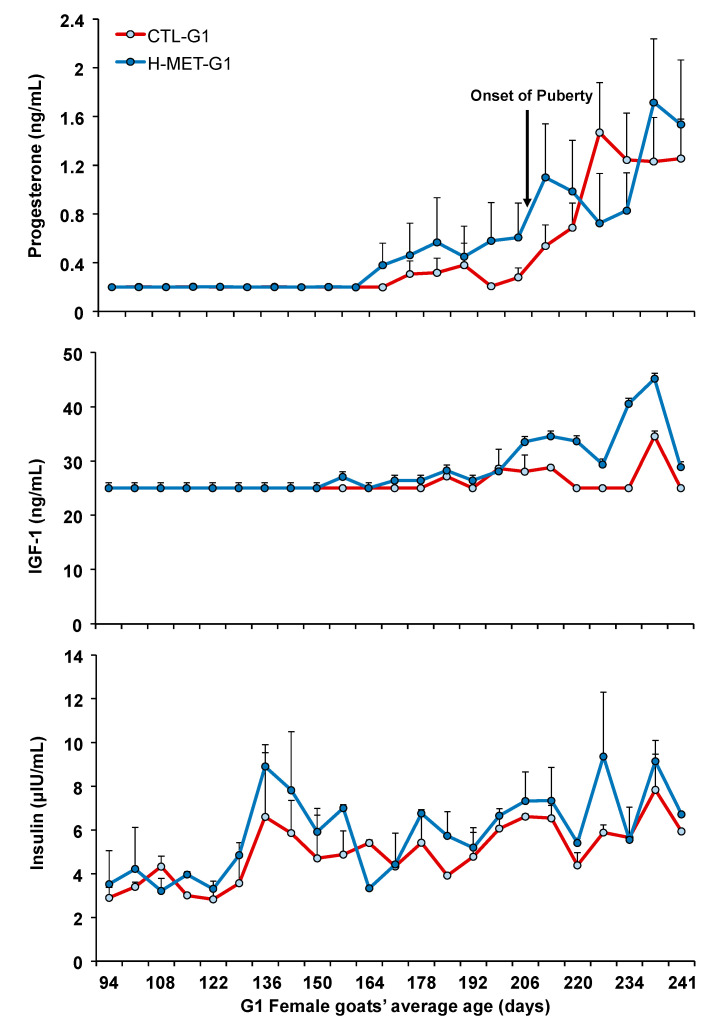
Plasma concentration of progesterone, IGF-1, and insulin of G1 doelings born to dams that received methionine supplementation (Optimethione^®^; H-MET-G0) or not (CTL-G0) during the last trimester of pregnancy. The black arrow indicates the onset of puberty. IGF-1 and insulin data represent pooled samples.

Plasma progesterone concentrations remained at basal levels from May (average age: 53 days) until late August (average age: 167 days), after which they began to increase similarly in both treatments, coinciding with the seasonal decrease in the natural photoperiod (Figure 3). Plasma progesterone concentrations did not differ between treatments (*p* > 0.05; Figure 3) and were not associated with live weight throughout the experiment (*p* > 0.05). The sampling date for plasma progesterone concentrations was significant across the experiment (*p* < 0.001).

**Figure 4:**
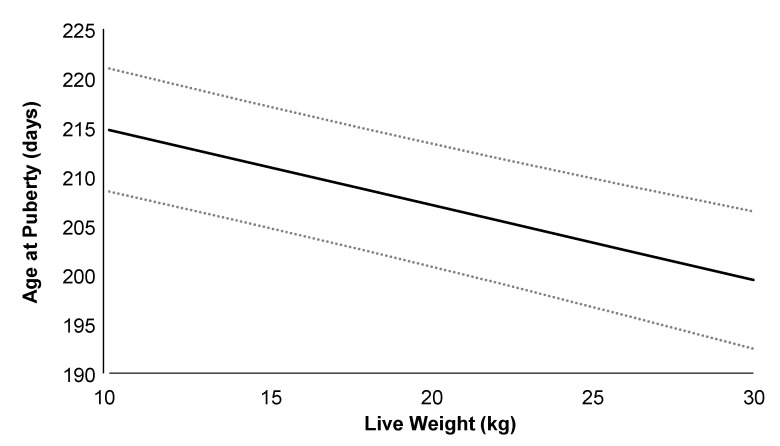
Relationship between age at puberty and live weight in G1 Alpine doelings born to dams that received (Optimethione^®^; H-MET-G0) or did not receive (CTL-G0) methionine supplementation in the last trimester of pregnancy. Data from H-MET-G1 and CTL-G1 are combined. The dashed lines represent upper and lower 95% confidence limits.

On average (±SD), the age at puberty onset was 223 ± 42 days, with a live weight of 20.8 ± 3.3 kg, corresponding to 60.0% of adult body weight. Doelings from H-MET-G1 achieved puberty at 216 ± 41 days, with a live weight of 20.5 ± 3.1 kg, representing 58.7% of their adult weight, whereas doelings from the CTL-G1 group reached puberty at 229 ± 42 days, with a live weight of 21.0 ± 3.5 kg, corresponding to 62.0% of their adult weight. Age and live weight at puberty did not differ significantly between treatments (*p* > 0.05; Table 2). Age at puberty was not positively associated with plasma total protein (*p* = 0.09), but it was associated with cholesterol (*p* < 0.01). The regression analysis estimated a 1.5-day increase in age at puberty for every 2.0 g/dL increase in plasma total protein. Puberty age was negatively associated with live weight (*p* < 0.001; Figure 4), plasma urea (*p* < 0.05), and glucose (*p* < 0.05) concentrations. The regression analysis estimated a 3.0-day decrease in age at puberty for every 4.0 kg increase in live weight.

Live weight at puberty was positively associated with plasma total protein concentrations (*p* < 0.001). The regression analysis estimated a 1.0 kg increase in live weight at puberty for every 2.0 g/dL increase in plasma total protein. Conversely, live weight at puberty was negatively associated with plasma urea concentration (*p* < 0.01). The regression analysis estimated a 0.3 kg increase in live weight at puberty for every 2.0 g/dL increase in plasma urea. Live weight at puberty was not associated with plasma cholesterol or glucose concentrations (*p* > 0.05). When data from all treatments were pooled for analysis, the correlation between puberty age and live weight was moderate and negative (*r*: 0.48; *p* < 0.001). A moderate and negative correlation was also observed between puberty age and circulating progesterone concentration (*r* = −0.51; *p* < 0.001). In contrast, moderate and positive correlations were found between puberty age and circulating concentrations of IGF-1 (*r* = 0.65; *p* < 0.001) and insulin (*r* = 0.52; *p* < 0.001).

### 3.3. G1 Age and Live Weight at Breeding and Conception

Age and live weight at the start of breeding did not differ between treatments (*p* > 0.05; Table 2). H-MET-G1 doelings conceived at an average age of 293 ± 16 days and a live weight of 22.5 ± 3.3 kg, while CTL-G1 doelings conceived at an average age of 298 ± 12 days and a live weight of 22.3 ± 2.1 kg. Age and live weight at conception did not differ significantly between treatments (*p* > 0.05; Table 2).

Age at conception was positively associated with plasma glucose (*p* < 0.01) and cholesterol (*p* < 0.001). The regression analysis estimated a 1.0 or 2.0-day increase in age at conception for every 20 mg/dL increase in plasma cholesterol or glucose, respectively. In contrast, age at conception was negatively associated with live weight (*p* < 0.001). The regression analysis estimated a 2.0-day decrease in age at conception for every 4.0 kg increase in total live weight. Age at conception was not influenced by live weight at the start of the breeding period (*p* > 0.05). Live weight at conception was not influenced (*p* > 0.05) by age at the beginning of the breeding period. Live weight at conception was positively associated (*p* < 0.001) with plasma total protein concentration. Live weight at conception increased by 1.4 kg when plasma total protein concentrations increased by 2.0 mg/dL. Additionally, live weight at conception was negatively associated with plasma urea concentrations (*p* < 0.01), with a decrease in 0.4 kg in live weight for every 20 mg/dL increase in plasma urea. However, live weight at conception was not associated with plasma cholesterol or glucose concentrations (*p* > 0.05).

For those who conceived, conception occurred at an average of 13 days after the start of the breeding period for H-MET-G1 doelings and 17 days for CTL-G1 does (*p* > 0.05; Table 2). A high proportion of CTL-G1 doelings (81%; 13 out of 16) and H-MET-G1 (66%; 6 out of 9) conceived during their first estrous cycle after the start of the breeding period. The remaining pregnant doelings conceived during their second estrus. The cycle of conception did not differ between treatments (*p* > 0.05)

Days to conception (DTC) were positively associated (*p* < 0.01) with plasma glucose and cholesterol (*p* < 0.001) concentration. The regression analysis estimated a 1.0 or 2.0-day increase in DTC for every 20 mg/dL increase in plasma cholesterol or glucose, respectively. DTC was negatively associated with live weight (*p* < 0.001). The regression analysis estimated a 2.0-day decrease in DTC for every 4.0 kg increase in total live weight. DTC was not associated with plasma total protein and urea concentrations (*p* > 0.05).

The correlations between breeding age and conception age (*p* < 0.01; *r*: 0.38) or DTC (*p* < 0.001; *r*: 0.44) were moderate and negative. The correlations between breeding age and circulating concentration of IGF-1 (*r*: 0.65) or insulin (*r*: 0.49) were moderate and positive (*p* < 0.001). The correlation between conception age and live weight was moderate and negative (*p* < 0.001; *r*: 0.74).

### 3.4. Reproductive Efficiency

The fertility rate was 53% (9 pregnant out of 17) for H-MET-G1 doelings and 76% (16 out of 21) for CTL-G1 doelings. There were no significant differences in fertility rates between treatments (*p* > 0.05; Table 3). Neither age nor live weight at the start of the breeding period influenced fertility rate (*p* > 0.05). Only one doeling from the H-MET-G1 treatment carried twins, while none from CTL-G1 group did. Prolificacy did not differ between treatments (*p* > 0.05; Table 3). Neither age nor live weight at the start of the breeding period had an effect on prolificacy (*p* > 0.05). The reproductive rate was 59% (10 fetuses) for H-MET-G1 doelings and 76% (16 fetuses) for CTL-G1 doelings. The reproductive rate did not differ between treatments (*p* > 0.05; Table 3). Neither age nor live weight at the start of the breeding period influenced the reproductive rate (*p* > 0.05).

### 3.5. Plasma Metabolites and Metabolic Hormones

**Figure 5:**
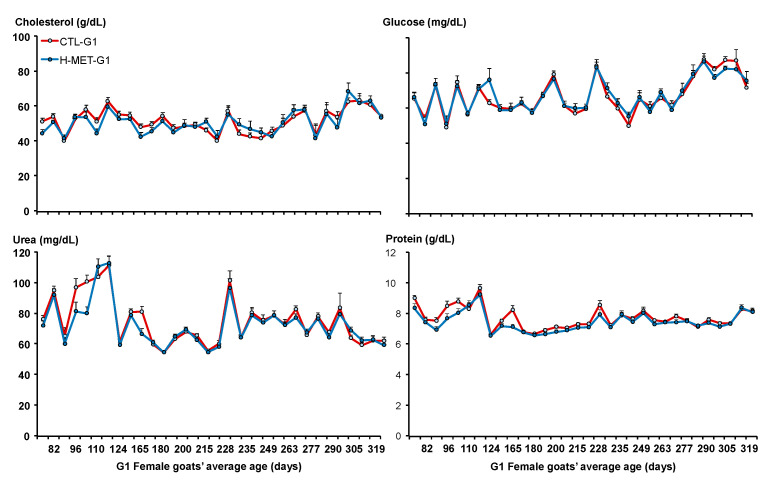
Plasma concentrations of cholesterol, glucose, urea, and protein of G1 doelings born to dams that received methionine supplementation (Optimethione^®^; H-MET-G0) or not (CTL-G0) during the last trimester of pregnancy.

Plasma glucose concentrations did not differ between treatments across the experimental period (*p* > 0.05; Figure 5). The sampling date was significant across the experiment (*p* < 0.001; Figure 5). Plasma glucose concentration was positively associated with live weight (*p* < 0.001). The regression analysis estimated a 2.8 mg/dL increase in plasma glucose for every 4.0 kg increase in live weight. Plasma cholesterol concentrations varied across the experiment and did not differ between treatments (*p* > 0.05; Figure 5). The sampling date was significant across the experiment (*p* < 0.001; Figure 5). Plasma cholesterol concentration was positively associated with live weight (*p* < 0.05). Plasma urea concentrations varied across the experiment and tended to differ between treatments (CTL-G1: 78.1 mg/dL vs. H-MET-G1: 74.5 mg/dL; *p* = 0.07; Figure 5). The sampling date was significant across the experiment (*p* < 0.001; Figure 4). Plasma urea concentration was not associated with live weight (*p* > 0.05).

Plasma cholesterol concentration was positively associated with live weight (*p* < 0.05). The regression analysis estimated a 2.9 mg/dL increase in plasma cholesterol for every 4.0 kg increase in live weight. Plasma total protein concentrations varied across the experiment but did not differ between treatments (*p* > 0.05; Figure 5). The sampling date was significant across the experiment (*p* < 0.001; Figure 4). Plasma cholesterol concentration was not associated with live weight (*p* > 0.05). Plasma IGF-1 concentrations varied across the experiment and differed between treatments (*p* < 0.001; Figure 3). Plasma insulin concentrations varied across the experiment and differed between treatments (*p* < 0.001; Figure 3). The sampling date was significant across the experiment for both variables (*p* < 0.001; Figure 3).

## 4. Discussion

We hypothesized that H-MET supplementation during late gestation (G0) would improve the metabolic profile, increase postweaning weight, accelerate puberty onset, and enhance reproductive efficiency in G1 offspring. Prenatal methionine supplementation showed no postnatal carryover effects. Growth, puberty age, and metabolic profiles were similar between groups, leading us to reject our hypothesis. We do not dismiss the potential benefits of H-MET supplementation in late gestation. However, the prenatal dose administered, the diet, and the compensatory growth observed in the females from the CTL-G1 group may have influenced our results.

### 4.1. G1 Postweaning Growth Pattern

We hypothesized that prenatal H-MET supplementation would increase postweaning growth patterns. Methionine supplementation has been shown to enhance mitochondrial function and energy metabolism; reduce oxidative stress; and increase growth factors, birth weight, and immune function in newborns [30,32,33,45,46]. In previous studies, newborns with higher birth weights were also heavier at weaning, suggesting that birth weight was positively correlated with weaning weight and indicating a postnatal carryover effect of prenatal methionine supplementation [30,32,33]. This effect is extended when nutritional restriction occurs, as prenatal methionine supplementation enhances intestinal function and increases birth and weaning weights [31,47]. Our results cannot confirm those results, as the weaning weight and growth patterns up to mating were similar between treatments. Although our diet was not restricted, it did not allow the maximum genetic expression for growth. When the diet is well-balanced, the effects of methionine are reduced and at minimum expression [48]. Nevertheless, prenatal methionine supplementation did not enhance growth patterns in Alpine female goats in our study.

### 4.2. G1 Doelings’s Onset of Puberty

Our second hypothesis was that prenatal H-MET supplementation would accelerate the onset of puberty. The fetal hypothalamus-pituitary gonadal (HPG) axis begins to function during mid-gestation, but its activity decreases during late pregnancy due to the suppressive effects of placental estrogens and androgens [49,50,51]. In sheep and goats, postnatal live weight and body composition are dominant factors in the onset of puberty by signaling the HPG axis about its metabolic status. Genetic selection and diet to maximize growth resulted in females attaining puberty sooner [18,52]. Given that females prenatally supplemented with methionine are heavier at birth and weaning [32,33,47], we expected similar results, such as enhanced growth performance; however, the growth pattern and age at the onset of puberty were similar between treatments.

Nevertheless, our results demonstrated a negative relationship between age at puberty and live weight, supporting the pivotal role of body composition on the onset of puberty. This result aligned with the increase in plasma insulin and IGF-1 concentration as puberty approached, which was previously reported [53,54]. Similarly, we observed a positive relationship between age at puberty, plasma total protein, and cholesterol concentrations. Cholesterol levels are closely related to metabolic status and modulate steroidogenesis [55,56]. Methionine supplementations can increase cholesterol concentrations [57]. In ewe lambs, a positive correlation was observed between the onset of puberty, plasma IGF-1 levels, and cholesterol concentrations [58]. Furthermore, glucose availability has a critical role in the onset of puberty, and we observed a negative relationship between age at puberty and plasma glucose and urea concentrations. The sensitivity of central sensors that detect the availability of particular plasma metabolites may change before the onset of puberty, as there is no relationship between plasma glucose, urea, and total protein levels and the onset of puberty [53,54,59].

Furthermore, our results indicated that doelings born in spring in subtropical zones could attain puberty in the same year. Spring-born females who experienced reduced physical activity or a well-balanced diet reached puberty in the same year [60,61]. However, this was not the case when their diet was limited or physical activity was increased [41]. As previously mentioned, our results indicated that increases in live weight reduced the age of puberty. Nevertheless, the progesterone levels remained flat until August, after which time this hormone began to rise, coinciding with the decrease in the natural photoperiod. Doelings in this experiment reached puberty at an average age of 223 d, 20.8 kg, and 60% of their adult live weight. In goat kids, puberty can be achieved when they reach 36% [62] or 50% [40,63] of their adult live weight. This result suggests that doelings may have accumulated sufficient body reserves to signal readiness for reproduction, as supported by the increased cholesterol, insulin, and IGF-1 levels leading to puberty. However, the photoperiod appears to be a stronger cue, delaying the onset of puberty until the days are shorter [64].

### 4.3. G1 Doeling’s Reproductive Efficiency

Heavier female goats at mating are more fertile and prolific [53,65,66]. Our results support this association, as we observed that age at conception was negatively related to live weight. Nevertheless, we expected that doelings born to dams that were H-MET supplemented would be heavier at mating and more fertile and prolific; however, weight at mating and reproductive efficiency, by increasing the number of pregnant females and fetuses in utero, were similar between treatments. Similarly, the days to conception were similar between treatments, and a higher proportion of does conceived during their first estrous cycle after the introduction of the buck. This indicates that pre-conceptional biostimulation synchronized the females, which supports previous results [42,62]. The similar reproductive performance between treatments could have been due to the postweaning diet offered, where goats gained, on average, 60 g/d. Goats have the ability for multiple ovulations and are highly adaptable to challenging environmental conditions. When food is scarce, they can adjust their metabolism and still get pregnant [67,68,69]. However, inadequate nutrition can reduce reproductive efficiency. For example, Saanen goats subjected to 19 days of severe energy deprivation experienced a decreased ovulation rate, although the proportion of goats coming into estrus remained unaffected [70]. As previously demonstrated, when the diet is restricted in quantity and quality, postnatal growth is delayed, affecting the desired weight at mating and jeopardizing reproductive efficiency [62,66,71]. By contrast, for doelings bred at nine months of age, the desired weight at mating is above 30 kg to achieve fertility rates above 85% [53,66,72]. Nevertheless, prenatal methionine supplementation did not have a carry-over effect, and the reproductive efficiency was similar between treatments. The pregnant females conceived on average at 22 kg; therefore, further analysis is necessary to determine the impact of kidding live weight and milk yield on their first lactation.

## 5. Conclusions

We concluded that prenatal herbal methionine supplementation did not enhance growth, puberty onset, or reproductive efficiency in Alpine doelings. Increased plasma insulin and IGF-1 concentrations toward the onset of puberty was observed. Both age at puberty and age at conception were negatively correlated with live weight but positively correlated with increased plasma concentrations of IGF-1 and insulin. A higher proportion of G1 doelings conceived during their first reproductive cycle after being biostimulated with teaser bucks. Future research should explore alternative doses of herbal methionine and strategies for optimizing maternal nutrition to improve offspring performance.

## Figures and Tables

**Table 1:** Nutrient composition (DM basis) of the diet offered and nutrient requirements for growing female goats (10 and 20 kg of body weight [39]).

**Ingredient Composition (% in Diet)**		
Alfalfa hay		70
Oat hay		10
Maize silage		20
**Diet Chemical Composition**		
Nutrient requirements/live weight	10 kg	20 kg
Dry matter intake (% live weight)	3.42	3.52
Crude protein (g)	38	57
Metabolizable energy (Mcal/d)	0.82	1.28
Calcium (g/d)	1.1	1.5
Phosphorus (g/d)	0.7	1.1
Dry matter intake (kg/doe/d)	0.26	0.54
Crude protein intake (g/doe/d)	22	51
Metabolizable energy intake (Mcal/doe/d)	0.57	0.96

**Table 2:** Age and live weight at the start of the breeding period and conception, and days to estrus after joining of doelings born to dams that received (Optimethione^®^; H-MET-G0) or did not receive (CTL-G0) methionine supplementation in the last trimester of pregnancy.

	Treatment			
	H-MET-G1	CTL-G1	SEM	*p*-Value
n	17	21		
Birth weight (kg)	3.4	3.0	0.06	0.004
Weaning Weight (kg)	9.2	8.2	0.28	0.10
LWG1 (g/d)	129	116	5.68	0.26
Age at puberty (d)	216	229	6.7	0.31
Weight at puberty (kg)	20.5	21	0.54	0.62
Age at breeding (d)	280	281	0.58	0.48
Weight at breeding (kg)	22.7	23.1	0.56	0.75
Age at conception (d)	293	298	2.72	0.55
LWG2 (g/d)	58	63	2.09	0.21
Weight at conception (kg)	22.5	22.3	0.51	0.84
Days to conception after joining	13	17	2.40	0.42

**Table 3:** Fertility rate (percentage of doelings pregnant per 100 doelings mated), litter size (number of fetuses per pregnant doeling), and reproductive rate (number of fetuses per 100 doelings exposed to fertile bucks) in doelings whose dams received (H-MET-G0) or did not receive (CTL-G0) methionine supplementation (Optimethione^®^) during the last trimester of pregnancy.

	Treatment		
	H-MET-G1	CTL-G1	*p*-Value
n	17	21	
Pregnancy (%)	53	76	0.15
Prolificacy (%)	11	0	0.97
Reproductive rate (%)	59	76	0.24

## Data Availability

The data presented in the manuscript were part of Diego Castillo-Gutierrez and Esau Ramírez-de la Cruz’s Honors Project. The corresponding author can provide information upon request.
